# High-throughput design and optimization of fast lithium ion conductors by the combination of bond-valence method and density functional theory

**DOI:** 10.1038/srep14227

**Published:** 2015-09-21

**Authors:** Ruijuan Xiao, Hong Li, Liquan Chen

**Affiliations:** 1 Beijing National Laboratory for Condensed Matter Physics, Institute of Physics, Chinese Academy of Sciences, Beijing 100190, China

## Abstract

Looking for solid state electrolytes with fast lithium ion conduction is an important prerequisite for developing all-solid-state lithium secondary batteries. By combining the simulation techniques in different levels of accuracy, *e.g.* the bond-valence (BV) method and the density functional theory (DFT), a high-throughput design and optimization scheme is proposed for searching fast lithium ion conductors as candidate solid state electrolytes for lithium rechargeable batteries. The screening from more than 1000 compounds is performed through BV-based method, and the ability to predict reliable tendency of the Li^+^ migration energy barriers is confirmed by comparing with the results from DFT calculations. β-Li_3_PS_4_ is taken as a model system to demonstrate the application of this combination method in optimizing properties of solid electrolytes. By employing the high-throughput DFT simulations to more than 200 structures of the doping derivatives of β-Li_3_PS_4_, the effects of doping on the ionic conductivities in this material are predicted by the BV calculations. The O-doping scheme is proposed as a promising way to improve the kinetic properties of this materials, and the validity of the optimization is proved by the first-principles molecular dynamics (FPMD) simulations.

Lithium-ion batteries (LIBs) are widely used in portable electronic devices^1^, hybrid and electric vehicles^2^, and they also show great potential application in the large-scale energy storage systems for intermittent power sources, such as wind or solar^3^. However, the liquid electrolytes used in current LIBs contain flammable organic solvents, leading to problems of leakage, vaporization, decomposition and safety^4^. One type of proposed next generation batteries is all-solid-state batteries, which are composed of both solid electrodes and solid electrolytes^5^. Because of the stability and non-flammability of inorganic solid electrolytes, the all-solid-state batteries are expected to exhibit less side reactions and higher safety. One of the biggest challenges for solid-state batteries is the development of good solid electrolytes. The high lithium ionic conductivity and the high electrical resistance are important prerequisites for solid electrolytes applicable to all-solid-state lithium secondary batteries, the former reduces the internal resistance of the battery and the later minimizes the self-discharge rate of the system^6^. The high electrical resistance can be realized in wide bandgap materials, conveniently predicted by electronic structure theory^7^. The investigations on fast lithium ion conductors are also widely performed^8^, but a comprehensive physical description is still not easy to grasp because the structure-properties correlations for ionic conductivity cannot be easily obtained^9^.

The approaches to understand ionic migration in solids start with space topology determined by the net channels in a specific crystalline structure^10^,^11^. This method is based on the hard geometric constrains in the atomic sublattice, from which a map of void, channel, and migration path is obtained by using the model of excluded volume and Voronoi-Dirichlet partition^11^,^12^,^13^. Although this method is rather vivid and intuitive, further studies indicate that the migration of lithium ions is not only determined by the geometrical-topological characteristics, since the interactions among atoms also plays an assignable role on the ion hopping^14^. A simple and available model to introduce the corresponding interactions is the bond-valence theory, in which the variation of the bond valence with the bond length reflects the actual softness of the interactions^15^,^16^,^17^,^18^. The bond-valence method is originally used to examine the stability of chemical structures or estimate the oxidation states of atoms^19^. The valence sum rule states that the sum of bond valences around any atom should be equal to the atomic valence^20^. According to this rule, the accessible sites for mobile ions can be obtained by analyzing the valence mismatch of moving ions, *i.e.*, the isosurface for a low enough valence mismatch threshold connecting equilibrium sites is considered as the continuous network for Li^+^ ion transport^6^,^18^,^21^,^22^. One of the disadvantages of the BV method is that no quantified value for energy barriers (*E*_a_) is obtained. Adams *et al.* linked the BV mismatch to the absolute energy scale and developed a novel BV-based force-field method by using a general Morse-type interaction potential^23^,^24^. Both the ion migration paths and energy barriers can be extracted from the energy landscape simulated by this energy-scaled BV method. The BV method is a fast technique, and the simulation of diffusion pathways and barriers for one crystal structure can be finished in several minutes by computer. The accuracy of the calculated energy barriers from the modified BV method is limited to the empirical potential energy function. Among energy models used in physics, chemistry and materials science, the quantum mechanical modelling method provides perhaps the most accurate description on the energy and electronic structures^25^. Therefore, the calculated energy barriers with higher reliability can be obtained from the transition-state method or the molecular dynamics method based on density function theory (DFT)^7^,^8^,^26^. However, they suffer from high computation cost which limits their efficiency on screening of materials based on ionic transport properties. Because of the distinctive features of each method, combination of the above approaches at different stage maybe a more practical scheme to discover solid electrolytes. The fast BV technique is suitable for high-throughput pre-screening a wide range of compounds since the trend in the ability of ion motion can be drawn from the relative values of the migration energy barriers despite of their less accuracy compared with quantum mechanical simulations. While the time-consuming DFT method can be adopted to do more precise calculations only for those promising candidates assigned by the BV method. For the derivative structures achieved by substitution or doping the existing compounds, the DFT computation is a powerful tool to predict exact structures which are important information for performing BV calculations. Thus, we believe that the reasonable combination of the BV method and DFT calculations is a great help in realizing the fast screening, design and optimizing solid state electrolytes for lithium secondary batteries.

In this work, employing the code implemented based on BV method, we calculate the lithium ion diffusion pathways and energy barriers for more than 1000 compounds from the structure database^27^. The effectiveness of the BV method is confirmed by comparing part of the results with DFT simulations. Besides screening a wide range of structures, the scheme combining BV and DFT method is also utilized to design and optimize the materials. We take β-Li_3_PS_4_, a promising electrolyte material identified in newly investigations^28^,^29^,^30^,^31^, as a model system, and the effects of doping at P site and S site on the structure and the ionic transport properties are investigated by DFT and BV method, respectively. As a result, an optimization scheme is proposed for this material.

## Results and Discussion

### BV lithium migration paths and barriers in LIB materials

**Figure 1:**
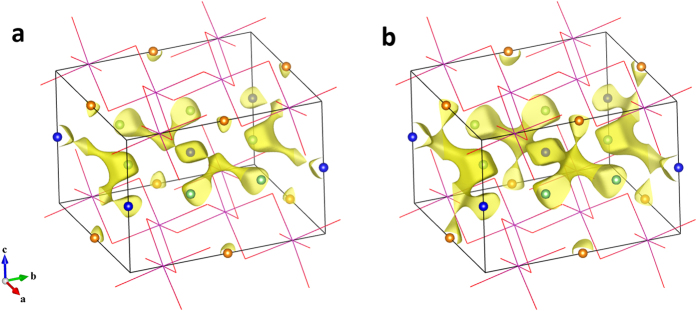
The isosurface of total potential energy *E*(Li). (**a**) The isosurface of *E*(Li) at 1.08 eV and (**b**) the isosurface of *E*(Li) at 1.55 eV for Li_2_MnO_3_ obtained by BV-based force-field method. The connected channels display the calculated lithium migration paths in this structure. The MnO_6_ framework is indicated as stick model, and the blue, green and orange bullets indicate Li at the 2c, 4 h and 2b site, respectively.

The transport pathways of Li ions calculated by BV-based force-field method are defined by the isosurface of total potential energy, *E*(Li). The critical isovalue to form a continuous channel in a certain direction reflects the migration energy barrier in this path, and the narrowest position along this way corresponds to the maximum energy image along this migration path, namely, the saddle point energy associated with a transition state. For a given compound, the different isovalues for connecting paths in various orientations displays the anisotropy diffusion in the structure. Figure 1 shows the calculated pathways of Li ions migrated in *ab* plane and along *c*-axis in layered Li_2_MnO_3_, an important component in Li-rich Mn-based high-capacity cathode^32^. In the structure of Li_2_MnO_3_, the Li ions distribute in both lithium layers and transition metal layers^33^. Previous DFT investigations reveal that the lithium layer is the main diffusion plane in this material, while the Li ions in the transition-metal layer also can diffuse in the structure by migrating into the lithium layer firstly^34^. The energy barrier for lithium migration in the lithium layers is found in the range of 0.54 ~ 0.84 eV, for the migration from the transition-metal layers to the lithium layers the barrier to be overcome is 0.73 ~ 0.80 eV^34^. The lithium migration pathways presented by BV-based method (Fig. 1) are qualitatively consistent with the DFT results. In Fig. 1(a), the isosurface of *E*(Li) = 1.08 eV, referring to the lowest potential energy found in the 3D grids of the cell, indicates the pathways between neighboring 4 h–4 h sites and those between 4 h-2c sites, both of which are within lithium layers. Further increasing the isovalue to *E*(Li) = 1.55 eV, the isosurface shown in Fig. 1(b) reveals the Li ion migration paths between adjacent lithium layer and transition-metal layer. The different threshold isovalue shows the minimum energies that must be overcome for Li ion migration for the two cases, and the lower energy barrier of the former path indicates that the Li ion transportation is much easier in lithium layers. For each hopping process, the Li ion migrates to the neighboring site along a curved path and the energy maximum in the path is near to the oxygen tetrahedral sites. These diffusion paths are same with those uncovered for the divacancy mechanism^35^ in DFT calculations. The straight paths appearing in the monovacancy mechanism^35^ are not found by BV-based method, because the lithium configuration is not considered in this calculation. In view of the above, although some information related to the distribution of Li ions is missing, the BV-based method still predict the proper lithium migration pathways compared with DFT simulations. The correct trend of activation energies can be achieved for these paths, confirming the effectiveness of the BV-based method in a certain structure.

**Figure 2:**
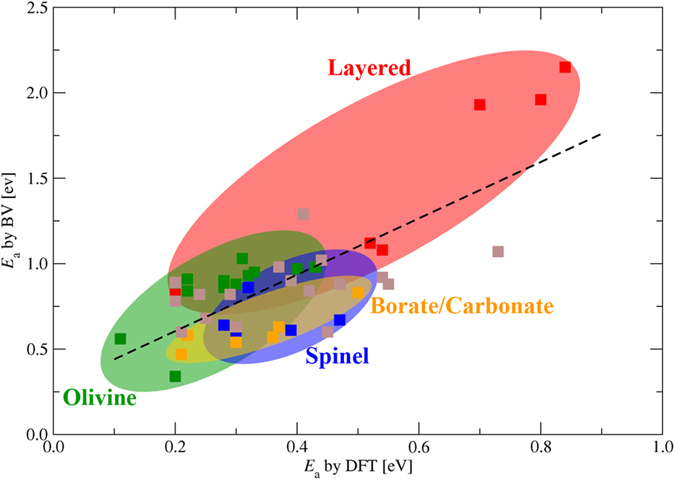
The calculated activation energies (*E*_a_) of lithium migration through BV-based method *vs*. the ones obtained by DFT method. The data symbol with red, green and blue color represents the compounds in layered-, olivine- and spinel-structure, respectively. The orange symbol represents the borate and carbonate, and the brown symbol displays the results for other types including phosphate, sulphate and various structures. The dash line is a linear fit for all the data here. The detailed information about the related compounds and the *E*_a_ values is listed in Table S1 of supplementary materials.

To screen materials according to the kinetic properties, we need to ensure the validity of the BV-based method in a much wider range of compounds. Partial match between the BV results and experimental measurements are observed. For example, the room temperature conductivity of the garnet-type Li_7_La_3_Zr_2_O_12_ (LLZO) with tetragonal structure is two orders of magnitude lower than its cubic modification^36^. Our calculated activation energies for these two phases are 1.05 eV and 0.96 eV, respectively, which correctly reflects the relative level of difficulty about Li ion diffusion. It should be noticed that the discrepancy between the calculated values and the experimental measurements exist because of the quantitative inaccuracy of the BV method caused by the adoption of empirical potential and the ignorance of the local distortion in the structure happened during lithium migration. However, the disagreements between experiments and BV prediction are also detected, *e.g.* the calculated Li ion migration barrier of Li_6_BaLa_2_Ta_2_O_12_ is 0.67 eV, lower than those of the two LLZO phases, while the experimental room temperature ionic conductivity of Li_6_BaLa_2_Ta_2_O_12_ is 4 × 10^−5^ S/cm^37^, locating between the value of cubic-LLZO (10^−4^ S/cm)^38^ and tetragonal-LLZO (10^−6^ S/cm)^39^. The experimental conductivities, including the contribution from bulk and grain boundary, are closely connected with the microstructures of samples, beyond the scope of current atomic-scale simulations. Thus, a more reasonable choice is to compare with a method also exploring the intrinsic self-diffusion properties of the bulk but with a higher level of accuracy. The energy barriers calculated through the first-principles molecular dynamics implicate the similar physical meaning with the activation energies of the BV-based approach, however, it is difficult to find large amounts of *E*_a_ values from the literatures because this method is computationally expensive. An alternate way is to use the *E*_a_ values calculated using DFT in combination with nudged-elastic band (NEB) method, which is an efficient way to find the minimal energy path between the initial and final state of a transition^40^ and has been applied a lot in both electrode and electrolyte materials. Figure 2 shows the comparison of the *E*_a_ values from the two methods. The calculated activation energies of lithium migration by BV-based method are plotted versus those by DFT simulations. There is a clearly consistent tendency between the predictions of these two methods since the data symbols are situated near to a line with positive slope. Besides the correct trend of the *E*_a_ values, the lithium diffusion pathways revealed by the BV method is also agreeable with those indicated by DFT method. For example, for all the layered structures considered here, the easiest migration way is along the 2D lithium layer; for olivine structures, the 1D path along a specific axis is shown; and the 3D connected channels are found in spinel structures; for borates and carbonates, the common feature in their structures is the existence of [BO_3_]^2−^ or [CO_3_]^2−^ groups, which are known to be trigonal planar. In a lot of cases, vacancies or interstitial sites can be found within or between the [BO_3_]^2−^ or [CO_3_]^2−^ layers. Take Li_2_CO_3_ as an example, in both the calculations by BV and DFT method, the role of the interstitial sites in the hopping processes is obviously observed. Although the consistent trend is found, it can also be noticed that the *E*_a_ value by BV method is always larger than that calculated by DFT calculations. This is because the relaxation of the structural frame during the lithium migration is ignored in the BV-based simulations. Accompanying with the movement of lithium ions, the adjustment of the positions of the anions and immobile cations, which composing the frame for lithium migration, occurs inevitably to release the local stress, lowering the total energy and stabilizing the system. This factor is included in the DFT calculations since each intermediate structure along the path is optimized according to the NEB method. However, it is completely neglected in the BV-based method in which the transition states are not considered. The difference mentioned above explains the data discrepancy between the two methods, nevertheless, the reliable tendency of the *E*_a_ values from BV-based method provides a qualitative assessment for the kinetic property evaluation among various compounds. Thus, we further apply the BV-based method to design the modification scheme for specific structures, taking β-Li_3_PS_4_ as an example, through combining it with DFT calculations in the next subsection.

### Doping optimization of β-Li_3_PS_4_

**Figure 3:**
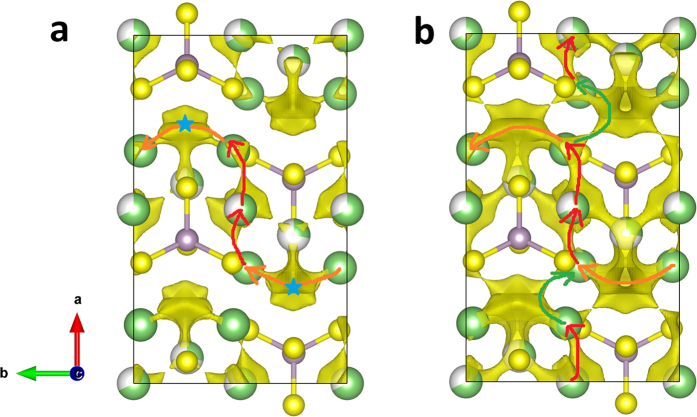
The BV-based method calculated lithium migration pathways for β-Li_3_PS_4_. (**a**,**b**) are the views of the isosurface of *E*(Li) = 0.79 eV and 1.01 eV perpendicular to *ab* plane, respectively. The green, gray and yellow bullets represent Li, P and S atomic sites, and the partial occupied lithium sites are indicated by partial filled green bullets according to the lithium concentration at the sites^31^. The blue stars in (**a**) represent the interstitial sites in the paths. The orange, red and green arrows indicate the connected diffusion channels.

Lithium thiophosphate compounds have been regarded as promising candidates for solid electrolytes because of their improved ionic conductivities relative to their oxygen isologs^41^. Among the ternary Li-P-S system, the most stable compound, Li_3_PS_4_, is reported to have a lithium ionic conductivity of 3 × 10^−7^ S/cm at 25 °C in its medium-temperature β phase^29^, higher than its low-temperature γ phase, and almost comparable to the commercial solid electrolyte material LiPON^42^. By microstructure design, the β phase can be stabilized at room temperature in the nanoporous structure and the ionic conductivity is further increased by 3 orders of magnitude^31^. The intrinsically high ionic conductivity is an indispensible factor for promoting the ionic conductivity in nanoporous β-Li_3_PS_4_, thus, increasing the kinetic property of the material itself is necessary. The migration of lithium ions in β-Li_3_PS_4_ has been investigated both experimentally^29^ and theoretically^43^. The experimental structural determination reveals that there are three inequivalent Li sites, 8d, 4b and 4c, in the crystal cell, and the latter two sites are 68% and 32% occupied, respectively^30^. Through DFT modelling, Lepley *et al.* found that the migration along *b* axis includes a lower energy configuration related to the interstitial site^43^, while the pathway along *a* axis is the most difficult one. The results from BV-based method as shown in Fig. 3(a) also indicates the existence of the interstitial sites (marked by blue stars in Fig. 3(a)) along the path from 8d to 8d site in *b* axis. The overall connected way along *b* axis is assisted by the hopping in *a* direction, that is, the channel existing in the chain of 8d-4b-8d, as indicated by red arrows in Fig. 3(a), through which the migration along *c* axis also can be realized. We notice that the zigzag pathway along the *c* axis reported in ref. 43 is in fact a series of hoping in alternate directions of *a* axis. Thus, the BV results agree with the general conclusions in previous DFT work^43^. What is different between our BV-based results and the reported DFT calculations^43^ is that we find different control step in *a* direction. To complete the whole migration along *a* axis, two types of hopping are needed. One is through 8d-4b-8d (indicated by red arrows in Fig. 3(a)), describing the Li^+^ movement within the PS_4_ layer, the other is directly from 8d to 8d (indicated by green arrows in Fig. 3(b)), which is the hopping of Li^+^ from one PS_4_ layer to another. In BV-based calculations, the interlayer migration channels are more difficult to form unless the isovalue is further increased to 1.01 eV, as displayed in Fig. 3(b), where additional connections are formed between 8d and 8d sites in *a* direction resulting in an entire coherent pathway going through the *a* axis. In ref. 43, the calculated energy barrier of the interlayer diffusion is 0.1 eV lower than that happens within the same PS_4_ layer, in a reverse order with our prediction. It should be noticed that different Li/vacancy configurations are adopted in our BV and Lepley’s DFT calculations. More comprehensive investigations on the kinetic properties of β-Li_3_PS_4_ are needed in the future work including the effect of Li/vacancy arrangements on the migration energy barriers. We also notice that the lithium migration pathways revealed in our calculations are similar with the diffusion channels deduced from the structural analysis on the basis of X-ray diffraction data^44^. Therefore, the consistency in general conclusions among different methods ensures the validity of the extension of the BV calculations into the doping design for β-Li_3_PS_4_.

A common feature in the structures of lithium thiophosphate is the plentiful existence of lithium interstitial sites or vacancies^45^, which provide a large stage to adjust their properties by changing the interstitial-vacancy character through doping or substitution. The concentration and distribution of lithium ions is affected by the substitution of P atoms for impurities with different valent state/atomic size, *e.g.* divalent (Zn), trivalent (Al and Ga), tetravalent (Si, Ge, Sn), and pentavalent (Sb). The replacement of S with O is also expected to influence the properties because of the different size between them. Thus, the effects of impurities at P sites and S sites on the crystal structures and ionic conductivities of β-Li_3_PS_4_ are analyzed by the combination of the DFT and BV methods. These compounds with the general formula, Li_3+x_P_1-y_M_y_S_4-z_O_z_ (M = Zn, Al, Ga, Si, Ge, Sn, Sb), are simulated in the unit cell of β-Li_3_PS_4_ with 4 PS_4_ groups. One M atom and one O atom are considered to put into the cell, which corresponds to y = 0 or 0.25, and z = 0 or 0.25, and (3 + x), the concentration of Li^+^, is determined according to the charge neutrality. The crystal lattice and ionic positions of the substituted cells are firstly optimized by the DFT calculations, which are followed by the study of ionic transport properties through BV-based method on the relaxed structures.

The arrangements of the solute atoms in the unit cell are dependent on the permutation and combination of Li/vacancy, P/M and S/O, and the number of all the configurations are more than tens of thousands. To reduce the possible arrangements and keep the computation cost at a reasonable level, we assume that Li atoms occupy the lattice sites in the order of 8d, 4b and 4c, which energetically go from low to high as determined by the DFT calculations^43^. The doping of M atom at P sites is considered at first, and the most stable arrangement of Li/vacancy and P/M is selected from all the possible configurations according to the DFT relaxation of these structures. For the co-doping scheme at both P and S sites, the solute structures are created based on the stable ones obtained for P site doping, that is the O atom replaces one of the S sites in the stable structure of Li_3+x_P_1-y_M_y_S_4_ obtained in the single-element doping case. In this way, the total number of arrangements of solute atoms selected for DFT calculations are 228, and the one with the lowest energy for each composition is determined as the most stable structure and used for the BVpath simulations in the next step.

**Figure 4:**
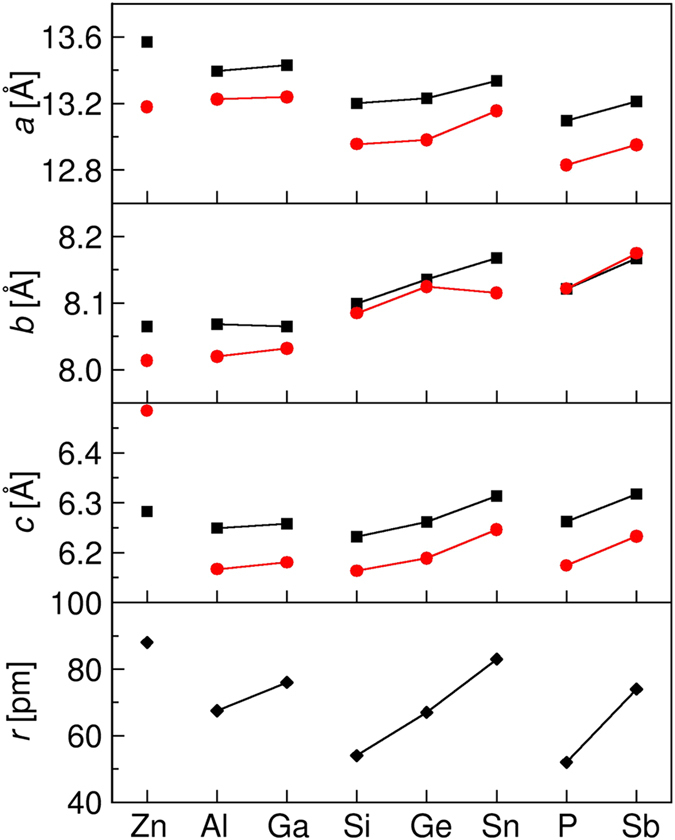
The calculated lattice constants *a*, *b* and *c* for the most stable structure of each doping case obtained by DFT simulations. The x-axis is labelled with the name of the element doped at P sites. The data represented by black squares and red circles are for the no doping at S sites and the O doping at S sites, respectively. The ionic radius of M ions are shown in bottom panel for comparison.

The doping causes the change of lattice parameters as shown in Fig. 4. For all the doping schemes, the lattice vectors are almost still keep orthogonal with the change of the angles between axes less than 1.1°. The trends of change in the lengths *a*, *b*, and *c* are mostly determined by the ionic radius of the doping elements, but the change is not isotropic and the variation in *a* direction is larger than that in *b* and *c* axis. This is because the interactions between Li^+^ and PS_4_ units are mainly distributed in *bc* plane, thus the loose bonding of Li-S pairs along *a* axis leaves less resistance for lattice deformation caused by ion substitution with larger ionic radius. For the co-doping case, the ion doping at P sites is larger than P^5+^ while the radius of O^2−^ is less than S^2−^ which make the change of lattice constants complex since it is related with both the ionic radii and the arrangement of M ions and O^2−^. The common feature is that doping at S sites with O^2−^ will result in lattice contraction in all three directions. The only exception happens in Li_3.75_P_0.75_Zn_0.25_S_3.75_O_0.25_, where the length of *c* axis is larger than the no doping structure Li_3.75_P_0.75_Zn_0.25_S_4_. Further analysis on the structural configurations indicates that the distance between Zn^2+^ and O^2−^ comes up to 8.1 Å, much larger than the values, around 5.0 Å, in other co-doping systems. The distinctive arrangement of solute atoms in Li_3.75_P_0.75_Zn_0.25_S_3.75_O_0.25_ leads to a more delicate change of the lattice compared to other cases.

**Figure 5:**
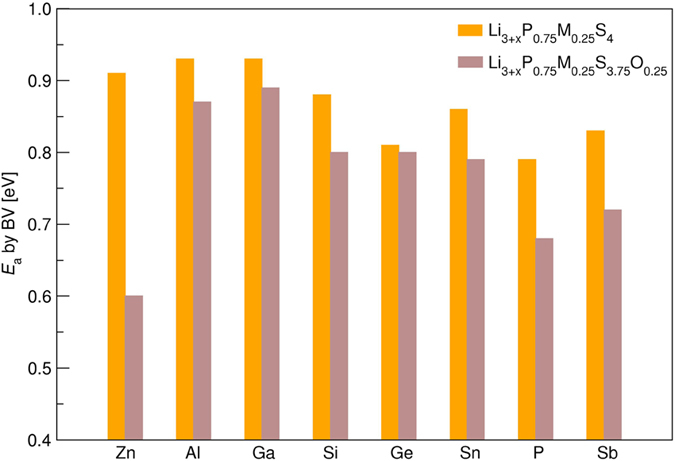
Lithium migration energy barriers, *E*_a_, calculated by force-based BV method for β-Li_3_PS_4_ and its doping derivatives.

**Figure 6:**
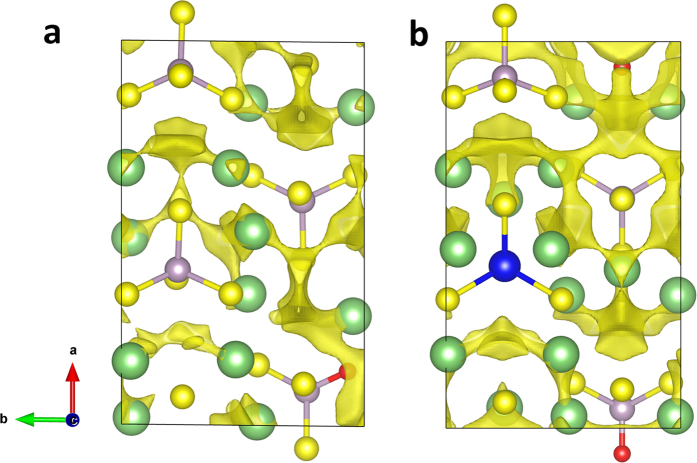
The lithium migration pathways revealed by BV-based method. (**a**) The Li^+^ pathway in β-Li_3_PS_3.75_O_0.25_ and (**b**) the Li^+^ pathway in β-Li_3.75_P_0.75_Zn_0.25_S_3.75_O_0.25_. The isosurfaces used to present the paths are *E*(Li) = 0.79 eV, as same as those used in Fig. 3. The green, gray, blue, yellow and red bullets represent Li, P, Zn, S and O atomic sites, respectively.

The deformation of the crystal lattice and the distortion of the local structure will affect the migration of Li^+^ ions since the diffusion activation energies, *E*_a_, are closely related with the void space and charge density in the unit cell. The calculated values of *E*_a_ by BV-based method are shown in Fig. 5 for all the most stable structures of each Li_3+x_P_1-y_M_y_S_4-z_O_z_ (y = 0 or 0.25, and z = 0 or 0.25) composition. As is known to us, the oxides generally present higher Li^+^ ion diffusion energy barriers than their sulfate analogs because of the weaker interactions between Li and S^41^. However, the simulations indicate that O doping at S sites can even reduce the values of *E*_a_ in some cases. One of the notable effects for O-doped β-Li_3_PS_4_ is that the migration energy barrier reduces from 0.79 eV for the primitive material to 0.68 eV for the doped system β-Li_3_PS_3.75_O_0.25_. The smallest *E*_a_ happens in the Zn and O co-doped structure β-Li_3.75_P_0.75_Zn_0.25_S_3.75_O_0.25_ with the value of 0.60 eV although the single Zn doping at P sites increases the *E*_a_ obviously. If ignoring the difference of hopping lengths, *a*, and frequencies, ν, in these structures, the estimated diffusion coefficient *D* will be raised by 3 ~ 4 orders of magnitude in β-Li_3_PS_3.75_O_0.25_ and β-Li_3.75_P_0.75_Zn_0.25_S_3.75_O_0.25_ compared with the pure material according to the equation, 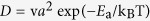. To understand the improvements of ionic conductivities caused by O doping, the lithium ion diffusion pathways are illustrated in Fig. 6 for the O doping and Zn, O co-doping structures. In Fig. 6(a), a connected channel is found around O atom, and a path along *a* axis is formed. This new transport path is not shown in Fig. 3(a), which changes the lithium migration mechanism of quasi-2D in β-Li_3_PS_4_ to 3-dimensional diffusion in its O doping structure β-Li_3_PS_3.75_O_0.25_. By looking into the local structural distortion caused by O doping, it can be found that the length of P-O bond is 1.58 Å, much smaller than P-S bond with the average length of 2.06 Å. However, the contraction of the unit cell caused by O-doping is only 2.03%, 0.01% and 1.42% in *a*, *b* and *c* axis, respectively. As a result, more space is left around O atom and provides wide channels for Li^+^ going through. Besides the kinetic properties, the electronic structures are also investigated. We take both the GGA and meta-GGA functionals in the DFT calculations since it is already known that the GGA functionals always underestimate the energy band gaps for many materials^46^. The modified Becker-Johnson exchange potential^47^ is chosen for the meta-GGA calculations. The band gaps obtained by GGA/meta-GGA are 2.81/4.01 eV and 2.91/4.12 eV for β-Li_3_PS_4_ and β-Li_3_PS_3.75_O_0.25_, respectively. The results indicate that O-doping at S site will not deteriorate the band gap of the system, ensuring high electrical resistance in the doped structure. For the Zn, O co-doping case, more paths along *a* axis are connected. The doping of Zn^2+^ with large ionic radius counterbalance the contraction of the unit cell introduced by O doping. In this lattice, the space left around O atom is even wider than that in the β-Li_3_PS_3.75_O_0.25_, thus the migration of Li^+^ along *a* axis becomes easier. We also notice that the lithium ions cannot go through the space around Zn^2+^ ion, because the lower charge of Zn^2+^ compared with P^5+^ will lead to weaker interaction between Zn and S and larger length of Zn-S bonds, blocking the paths around it. The similar results happen in the single-element doping at P sites. In the substitutions of P^5+^ ion with Zn^2+^, Al^3+^, Ga^3+^, Si^4+^, Ge^4+^, Sn^4+^ or Sb^5+^, none of them shows smaller migration energy barrier than primitive β-Li_3_PS_4_, thus the doping at P sites is not expected to improve the kinetic properties of this material.

### FPMD simulations of β-Li_3_PS_4_ and derivatives

**Figure 7:**
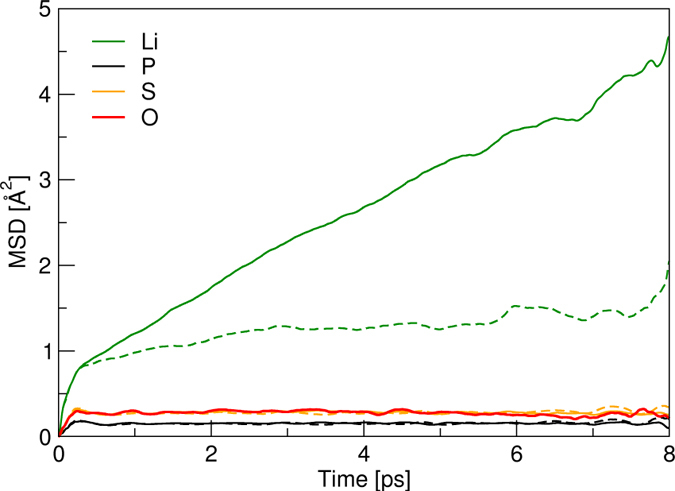
Mean square displacements (MSDs) by the FPMD simulations at 600 K. The MSDs of Li, P, S, and O atoms are presented by dashed lines for β-Li_3_PS_4_ and by solid lines for β-Li_3_PS_3.75_O_0.25_.

To confirm the better ionic transport appeared in O-doped β-Li_3_PS_4_ revealed by BV-based method, the FPMD simulations are carried out to further demonstrate the effects of O-doping at S sites on the kinetic properties of the system. The MD simulations are ran for both β-Li_3_PS_4_ and β-Li_3_PS_3.75_O_0.25_ for 8000 steps with time step of 1 fs at 600 K, following an equilibrium process of 2 ps at this temperature. A common measure in MD calculations used to characterize the diffusion behavior is the mean square displacement (MSD), expressed as 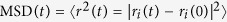, where *r*_*i*_(t) is the position of the *i*-th ion at the time *t*, and the average is over the time steps and all the ions. Figure 7 shows the MSD for Li, P, S and O atoms in the primitive structure and the O-doping case. For both structures, the instantaneous MSD for P and S(O) atoms have constant values near to zero, indicating that P and S(O) atoms oscillate with respect to their original equilibrium positions to keep the lattice frame of the material. However, the MSD for Li ions show obvious difference in two structures. The MSD of Li ions in β-Li_3_PS_4_ looks more or less flat around 1 Å^2^, corresponding to the mean travel distance of ~1 Å, less than half of the distance between the nearest neighboring Li-Li sites, 2.52 Å. Therefore, the Li ions in β-Li_3_PS_4_ averagely fail to complete the hopping from one crystal site to another. Looking into the MSD of each Li ion gives more details about the movement of the ions. Analysis indicates that only two hoppings are completed during the simulation time, one is the migration indicated by orange arrows in Fig. 3(a), which is the movement from 8d to 8d site along *b* axis, the other is the Li^+^ hopping from 8d to 4b in *a* direction, which is the diffusion of Li^+^ within PS_4_ layers. The paths revealed by FPMD simulations are consistent with our BV-based force-field calculations. Several Li^+^ ions move to the neighboring interstitial sites and part of them come back to their original positions at the end of the simulation. Most of Li^+^ ions start moving but only vibrate around their equilibrium sites, which is similar to the behavior of P and S atoms except with much larger amplitudes. In contrast, the MSD of Li ions in β-Li_3_PS_3.75_O_0.25_ becomes a linear function of time, and at the end of the simulation time the migration distance of Li ions is close to the length of neighboring Li-Li pair, indicating the diffusion of Li ions from one atomic site to another. In other words, the Li^+^ ions in β-Li_3_PS_3.75_O_0.25_ migrate much easier than those in β-Li_3_PS_4_, consistent with the prediction given by BV-based calculations, and further confirming that O-doping is a promising optimization scheme to improve the kinetic properties of the β-Li_3_PS_4_. Interestingly, Takada *et al.* reported the enhancement of ionic conductivity in quenched (1-*x*)Li_3_PS_4_–*x*Li_3_PO_4_ composites and identified the β phase and another metastable phase in the products^48^. We have focused our experiments on the predicted β-Li_3_PS_3.75_O_0.25_ crystal phase and its kinetic properties.

## Conclusion

We report a design scheme of fast lithium ion conductors as candidate solid state electrolytes for lithium rechargeable batteries through combining simulation methods with different accuracy, *e.g.* the bond-valence method and density functional theory. The BV technique, fast but less accurate, is a powerful tool for high-throughput pre-screening a wide range of compounds because the trend of migration energy barriers can be correctly predicted. The expensive DFT method is suitable for more precise calculations for those promising candidates or derivative structures. The effectiveness of the combination method is demonstrated in this work. By implementing the computer code of BV-based force-field approach, we realize the screening of solid state electrolytes from more than 1000 compounds in the structure database. The BV method reveals lithium ion pathways in *ab* plane and along *c*-axis in the layered-cathode Li_2_MnO_3_, consistent with the DFT results, confirming the validity of the BV-based method in a certain structure. Further comparison between the activation energies obtained from the BV-based technique and the DFT simulations for nearly 50 compounds proves that the reliable tendency of the energy barriers from BV method is a valid qualitative assessment for the kinetic property among various structures. The application of the combination method is exampled in the fast optimization of β-Li_3_PS_4_ system. The high-throughput DFT simulations are adopted to give the relaxed structures of the doping derivatives of β-Li_3_PS_4_, including the substitution of P atoms for impurities with divalent (Zn), trivalent (Al and Ga), tetravalent (Si, Ge, Sn), and pentavalent (Sb) ions, and the replacement of S with O element. Following the DFT relaxations, the effects of doping on the ionic conductivities of β-Li_3_PS_4_ are predicted by the BV method, which indicates that P-site doping is not expected to decrease the lithium migration energy barriers, while the O-doping at S site improves the kinetic properties obviously. Local structure analysis explains easier hopping of Li^+^ around O atom because of the wider space around the channel near to O compared with that around S. The results from FPMD simulations for β-Li_3_PS_4_ and β-Li_3_PS_3.75_O_0.25_ confirms that the O-doping is a promising optimization scheme to improve the ionic transportation in β-Li_3_PS_4_. In summary, the reasonable combination of the BV method and DFT calculations is effective to realize the fast screening, design and optimizing solid state electrolytes for lithium secondary batteries.

## Methods

### BV-based force-field calculations

The BV calculations are performed using the computer code, BVpath, which is implemented by ourselves. The code is built upon the BV-based force-field approach proposed by Adams *et al.*^24^. Both the interaction energy in Morse-type potential and the Coulombic repulsion are calculated on 3D grids. The former describes the attraction between a dummy lithium ion at the grid and the anions around it, and the latter is the electrostatic repulsion term between the immobile cations and the dummy ion. The summation of above two terms on grids of points builds the maps of the total potential energy, *E*(Li), in a unit cell, based on which the regions enclosed by isosurfaces of constant *E*(Li) can be obtained. The enclosed channels are considered as the space where lithium ions can go through, and the threshold value of *E*(Li) to form a continuous pathway is estimated as the Li^+^ migration energy barrier. The cutoff radius of 10 Å for the interaction model is taken according to the suggestion from Adams and Rao^24^. The grid resolution of 0.1 Å is used for the calculations, and the convergence is confirmed by comparing with the results of 0.05 Å for 10 testing structures. For oxide compounds, we take the parameters characterizing Morse-type potential given in ref. 24. To cover a broader range of materials in the study, we derived the Morse potential parameters for lithium and other anions from the softBV model^49^. The parameters are listed in Table 1. The charges used to evaluate the Coulombic repulsion is estimated according to the equation 8 in ref. 24. The BVpath code is written by Fortran and the calculation time for a single structure is a few minutes. To realize the automatic flow for simulations of a large database of structures, a set of scripts written in the Python programming language are developed to generate input files from the information in structure database and to perform a high-throughput computation. During the post-calculation analysis, the isosurfaces of potential energy *E*(Li) is shown by VESTA visualization package^50^.

### DFT calculations

For the compounds with the lack of complete structure information, DFT is a reliable method to predict the lattice parameters and atomic sites since an exact and precise structure is an important factor to ensure the effectiveness of the BV calculations. This is the case for the new structures derived by substitution or doping from a promising parent compound. Instead of screening the materials, the structural relaxation performed by the DFT method plays a role of pretreatment for the simulation of ionic transport through the BV approach. The DFT calculations are performed with the Vienna *ab initio* simulation package (VASP)^51^ using the generalized gradient approximation (GGA) with a parameterized exchange-correlation functional according to PBE^52^. The cutoff for the wave function is specified as 30% larger than the maximal cutoff value among all elements involved in the compound. The k-mesh used to sample the Brillouin zone is in the density of one point per 0.06 Å^−3^ and the gamma point is included. Both ions and cells are relaxed in the optimization with the energy and force convergence criterion of 10^−5 ^eV and 0.01 eV/Å, respectively. Scripts to control the automatic simulation flow are also developed for high-throughput DFT calculations. The primary role of these scripts is to initiate the calculations, including creating the derived structures, generating input files and specifying the necessary parameters. They also detect the errors and return the message if the DFT calculations for the structural relaxation are terminated abnormally. In the part of optimal design of electrolytes based on β-Li_3_PS_4_ structure, to further confirm the kinetic properties revealed by BV method, the first-principle molecular dynamics (FPMD) simulations based on density functional theory are performed for this material and its derivative. The FPMD run is carried out on a supercell with 16 formula units (f.u.). A Nose thermostat is adopted for the simulations with the temperature of 600 K. The FPMD calculations are lasted for 10000 steps with a time step of 1 fs, and only the Γ point is used for the Brillouin zone sampling. The calculation for the first 2 ps is used to equilibrate the system at the temperature, and the migration pathways and transport properties of Li ions are extracted from the last 8000 steps.

## Additional Information

**How to cite this article**: Xiao, R. *et al.* High-throughput design and optimization of fast lithium ion conductors by the combination of bond-valence method and density functional theory. *Sci. Rep.* **5**, 14227; doi: 10.1038/srep14227 (2015).

## Supplementary Material

Supplementary Information

## Figures and Tables

**Table 1:** The BV parameters *b*, *R*_0_, cutoff radius *R*_co_, and the relative bond softness |*σ*_X_-*σ*_A_|, taken from ref. 32.

anion	*b*[Å]^32^	*R*_0_[Å]^32^	*R*_co_ [Å]	|*σ*_X_-*σ*_A_|^32^	*N*_c_	*R*_min_ [Å]	*D*_0_ [eV]
O(−2)	0.516	1.17096	6	0.11905	5.021	1.94001	0.98816
S(−2)	0.653	1.46652	6	0.19586	4.604	2.40971	1.04027
Se(−2)	0.684	1.62021	7	0.21320	5.118	2.68386	0.88750
Te(−2)	0.729	1.71028	7	0.23869	4.333	2.73313	0.88544
F(−1)	0.508	1.08674	6	0.11415	5.289	1.87265	0.49611
Cl(−1)	0.634	1.39892	6	0.18542	5.258	2.39647	0.49302
Br(−1)	0.675	1.51288	7	0.20822	5.263	2.58255	0.44910
I(−1)	0.735	1.64829	7	0.24210	5.000	2.78807	0.44117

The average cation coordination number *N*_c_ is obtained by gathering statistics with the lithium ion coordination numbers in compounds found in structure database. Based on the BV parameters and the value of *N*_c_, the Morse potential parameters, energy minimum distance *R*_min_ and dissociation energy *D*_0_, are derived according to the BV-based force-field model.
