# Supplementary material for: High-throughput design and optimization of fast lithium ion conductors by the combination of bond-valence method and density functional theory
Source: Sci Rep. 2015 Sep 21;5:14227. doi: 10.1038/srep14227 (PMC4585693; doi:10.1038/srep14227)
Supplement: Supplementary Information [file srep14227-s1.pdf]

SUPPLEMENTARY INFORMATION FOR

High-throughput design and optimization of fast lithium ion conductors by the combination of bond-valence method and density functional theory

Ruijuan Xiao*, Hong Li and Liquan Chen

Key Laboratory for Renewable Energy, Beijing Key Laboratory for New Energy Materials and Devices, Beijing National Laboratory for Condensed Matter Physics, Institute of Physics, Chinese Academy of Sciences, Beijing 100190, China

*corresponding author: R. X. (rjxiao@iphy.ac.cn)

Table S1. The activation energies (E_a) of lithium migration calculated by BV-based method and DFT method for 46 compounds.

No.	Compounds	E_a by DFT	E_a by BV	Comments
1	$LiMnPO_4$	0.31[1]	1.03	Olivine[2]
2	$LiFePO_4$	0.32[1]	0.93	Olivine[3]
3	$LiCoPO_4$	0.28[1]	0.90	Olivine[4]
4	$LiNiPO_4$	0.22[1]	0.91	Olivine[2]
5	$LiMgPO_4$	0.30[5]	0.88	Olivine[6]
6	$LiAlSiO_4$	0.20[5]	0.34	Olivine[7]
7	$LiScSiO_4$	0.33[5]	0.95	Olivine[9]
8	$LiInSiO_4$	0.40[5]	0.97	Olivine[10]
9	$LiTmSiO_4$	0.43[5]	0.98	Olivine[11]
10	$LiAlGeO_4$	0.11[5]	0.56	Olivine[12]
11	$LiInGeO_4$	0.28[5]	0.86	Olivine[13]
12	$LiScGeO_4$	0.22[5]	0.84	Olivine[14]
13	$LiMn_2O_4$	0.39[15]	0.61	Spinel[16]
14	$LiNi_{1/2}Ge_{3/2}O_4$	0.47[17]	0.67	Spinel[18]
15	$LiNi_{1/2}Mn_{3/2}O_4$	0.32[17]	0.86	Spinel[19]
16	$LiTi_2O_4$	0.28[20]	0.64	Spinel[21]
17	$Li_4Ti_5O_{12}$	0.30[22]	0.59	Spinel[23]
18	$LiCoO_2$	0.84[24]	2.15	Layer[25]
19	$LiCrO_2$	0.80[26]	1.96	Layer[27]
20	Li_2MnO_3	0.54[28]	1.08	Layer[29]
21	Li_2IrO_3	0.52[30]	1.12	Layer[31]
22	$LiTiS_2$	0.70[32]	1.93	Layer[33]
23	$LiFeSO_4OH$	0.20[34]	0.82	Layer[35]
24	$LiMnBO_3$	0.36[36]	0.57	Borate/Carbonate[37]
25	$LiFeBO_3$	0.22[36]	0.58	Borate/Carbonate[38]
26	$LiCoBO_3$	0.37[36]	0.63	Borate/Carbonate[39]
27	$LiBO_2$	0.50[40]	0.83	Borate/Carbonate[41]
28	$Li_{2.6}C_{0.33}B_{0.67}O_3$	0.21[42]	0.47	Borate/Carbonate[42]
29	Li_2CO_3	0.30[43]	0.54	Borate/Carbonate[44]
30	Li_3OCl	0.37[45]	0.98	Antiperovskite[46]
31	Li_3OBr	0.41[45]	1.29	Antiperovskite[46]
32	β-Li_3PO_4	0.39[47]	0.90	Phosphate[48]
33	γ-Li_3PO_4	0.29[47]	0.82	Phosphate[49]
34	β-Li_3PS_4	0.20[50]	0.78	Thiophosphate[51]
35	$Li_7P_3S_{11}$	0.24[52]	0.82	Thiophosphate[53]
36	$Li_2Fe(SO_4)_2$	0.47[54]	0.88	Sulphate[55]
37	$Li_2Mn(SO_4)_2$	0.54[54]	0.92	Sulphate[56]
38	$Li_2Co(SO_4)_2$	0.42[54]	0.84	Sulphate[55]
39	$LiAlPO_4F$	0.55[57]	0.88	Tavorite[58]

40	$LiMgSO_4F$	0.20[57]	0.89	Tavorite[59]
41	$LiFeSO_4F$	0.44[60]	1.02	Tavorite[61]
42	$FeSO_4F$	0.21[62]	0.60	Tavorite[62]
43	$LiTi_2(PO_4)_3$	0.25[63]	0.68	NASICON[64]
44	Li_2NiO_2	0.45[65]	0.60	Orthorhombic[66]
45	Li_2O	0.30[67]	0.63	Cubic[68]
46	LiF	0.73[69]	1.07	Cubic[70]

References

1. Kutteh, R. & Avdeev, M. Initial Assessment of an Empirical Potential as a Portable Tool for Rapid Investigation of Li^+ Diffusion in Li^+-Battery Cathode Materials. *J. Phys. Chem. C* **118**, 11203-11214 (2014).
2. García-Moreno, O. *et al.* Influence of the Structure on the Electrochemical Performance of Lithium Transition Metal Phosphates as Cathodic Materials in Rechargeable Lithium Batteries: A New High-Pressure Form of $LiMPO_4$ (M = Fe and Ni). *Chem. Mater.* **13**, 1570-1576 (2001).
3. Rousse, G., Rodriguez-Carvajal, J., Patoux, S. & Masquelier, C. Magnetic Structures of the Triphylite $LiFePO_4$ and of Its Delithiated Form $FePO_4$. *Chem. Mater.* **15**, 4082-4090 (2003).
4. Kubel, F. Crystal structure of lithium cobalt double orthophosphate, $LiCoPO_4$. *Z. Kristallogr.* **209**, 755-755 (1994).
5. Jalem, R., Nakayama, M., & Kasuga, T. An efficient rule-based screening approach for discovering fast lithium ion conductors using density functional theory and artificial neural networks. *J. Mater. Chem. A* **2**, 720-734 (2014).
6. Hanic, F., Handlovic, M., Burdova, K. & Majling, J. Crystal structure of lithium magnesium phosphate, $LiMgPO_4$: Crystal chemistry of the olivine-type compounds. *J. Cryst. Spectrosc. Res.* **12**, 99-127 (1982).
7. Ceriani, C., Fois, E. & Gamba, A. The role of extra-framework cations on the structure of dehydrated Li-ABW. A computer simulation study. *Microporous Mesoporous Mater.* **57**, 73-81 (2003).
8. Blasse, G. & Bril, A. Structure and Eu^{3+}-fluorescence of lithium and sodium lanthanide silicates and germinates. *J. Inorg. Nucl. Chem.* **29**, 2231-2241 (1967).
9. Steele, I. M., Pluth, J. J. & Ito, J. Crystal structure of synthetic $LiScSiO_4$ olivine and comparison with isotypic Mg_2SiO_4. *Zeitschrift für Kristallographie - Crystalline Materials* **147**, 119-127 (1978).
10. Redhammer, G. J. & Roth, G. $LiInSiO_4$: a new monovalent-trivalent olivine. *Acta Crystallographica C* **59**, 38-40 (2003).
11. Kono, Y., Uematsu, X. & Sato, M. Structural analysis and electrical property of Li-Ln-Si oxide systems. *Kidorui* **24**, 148-149 (1994).
12. Tripathi, A., Kim, S. J., Johnson, G. M. & Parise, J. B. Synthesis and single-crystal structure of a lithium aluminogermanate with the zeolite ABW topology. *Microporous Mesoporous Mater.* **34**, 273-279 (2000).
13. Touboul, M. & Toledano, P. Structure du germanate d`indium et de lithium. *Acta*

Crystallographica C **43**, 2004-2006 (1987).

14. Genkina, E. A., Timofeeva, V. A. & Bykov, A. B. Structure of the Olivine-type Li, Sc-germanate $LiScGeO_4$. *J. Struct. Chem.* **27**, 167-168 (1986).
15. Nakayama, M., Kaneko, M. & Wakihara, M. First-principles study of lithium ion migration in lithium transition metal oxides with spinel structure. *Phys. Chem. Chem. Phys.* **14**, 13963-13970 (2012).
16. Berg, H., Thomas, J. O., Wen, L. & Farrington, G. C. A neutron diffraction study of Ni substituted $LiMn_2O_4$. *Solid State Ionics* **112**, 165-168 (1998).
17. Nakayama, M., Jalem, R. & Kasuga, T. Electronic structure of spinel-type $LiNi_{1/2}Ge_{3/2}O_4$ and $LiNi_{1/2}Mn_{3/2}O_4$ as positive electrodes for rechargeable Li-ion batteries studied by first-principles density functional theory. *Solid State Ionics* **262**, 74-76 (2014).
18. Hirota, K., Ohtani, M., Mochida, N. & Ohtsuka, A. Formation and structure of spinel solid solution in Li_2O - MO - GeO_2 (M=Zn,Co,Ni) system. *J. Ceram. Soc. Jpn.* **96**,92-96 (1988).
19. Gryffroy, D., Vandenberghe, R.E., Legrand, E. A Neutron Diffraction Study of some Spinel Compounds Containing Octahedral Ni and Mn at a 1:3 Ratio. *Mater. Sci. Forum* **79**, 785–790 (1991).
20. Bhattacharya, J. & Van der Ven, A. Phase stability and nondilute Li diffusion in spinel $Li_{1+x}Ti_2O_4$. *Phys. Rev. B* **81**, 104304 (2010).
21. Cava, J. R., Murphy, D. W. & Zahurak, S. M. The crystal structures of the lithium-inserted metal oxides $Li_{0.5}TiO_2$ anatase, $LiTi_2O_4$ spinel, and $Li_2Ti_2O_4$. *J. Solid State Chem.* **53**, 64-75 (1984).
22. Ziebarth, B., Klinsmann, M., Eckl, T. & Elsässer, C. Lithium diffusion in the spinel phase Li4Ti5O12 and in the rocksalt phase $Li_7Ti_5O_{12}$ of lithium titanate from first principles. *Phys. Rev. B* **89**, 174301(2014).
23. Deschanvres, A., Raveau, B. & Sekkal, Z. Mise en evidence et etude cristallographique d'une nouvelle solution solide de type spinelle $Li_{1+x}Ti_{2-x}O_4$ $0 \leqslant x \leqslant 0.33$. *Mater. Res. Bull.* **6**, 699-704 (1971).
24. Van der Ven, A., Ceder, G., Asta, M. & Tepesch, P. D. First-principles theory of ionic diffusion with nondilute carriers. *Phys. Rev. B*, **64**, 184307 (2001).
25. Holzapfel, M., Haak, C. & Ott, A. Lithium-Ion Conductors of the System $LiCo_{1-x}Fe_xO_2$, Preparation and Structural Investigation. *J. Solid State Chem.* **156**, 470-479 (2001).
26. Lyu, Y. C. *et al.* Atomic insight into electrochemical inactivity of lithium chromate ($LiCrO_2$): Irreversible migration of chromium into lithium layers in surface regions. *J. Power Sources* **273**, 1218-1225 (2015).
27. Lu, Z.-H. & Dahn, J. R. Structure and Electrochemistry of Layered $Li[Cr_xLi_{(1/3-x/3)}Mn_{(2/3-2x/3)}]O_2$. *J. Electrochem. Soc.* **149**, A1454-1459 (2002).
28. Xiao, R. J., Li, H. & Chen, L. Q. Density Functional Investigation on Li_2MnO_3. *Chem. Mater.* **24**, 4242-4251 (2012).
29. Strobel, P. & Lambert-Andron, B. Crystallographic and magnetic structure of Li_2MnO_3. *J. Solid State Chem.* **75**, 90-98 (1988).
30. Chen, Y. C., Huo, M., Song, L. J. & Sun, Z. L. Electrical and lithium ion dynamics in Li_2IrO_3 from density functional theory study. *RSC Adv.* **4**, 42462-42466 (2014).
31. O'Malley, M. J., Verweij, H. & Woodward, P. M. Structure and properties of ordered Li_2IrO_3 and Li_2PtO_3. *J. Solid State Chem.* **181**, 1803-1809 (2008).
32. Van der Ven, A., Thomas, J. C. & Xu, Q. C. Nondilute diffusion from first principles: Li diffusion

in Li_xTiS_2. *Phys. Rev. B* **78**, 104306 (2008).

33. Dahn, J. R., McKinnon, W. R., Haering, R. R., Buyers, W. J. L. & Powell, B. M. Structure determination of Li_xTiS_2 by neutron diffraction. *Can. J. Phys.* **58**, 207-213 (1980).
34. Eames, C., Clark, J. M., Rousse, G., Tarascon, J.M. & Islam, M. S. Lithium Migration Pathways and van der Waals Effects in the $LiFeSO_4OH$ Battery Material. *Chem. Mater.* **26**, 3672-3678 (2014).
35. Subban, C. V. *et al.* Preparation, Structure, and Electrochemistry of Layered Polyanionic Hydroxysulfates: $LiMSO_4OH$ (M = Fe, Co, Mn) Electrodes for Li-Ion Batteries. *J. Am. Chem. Soc.* **135**, 3653-3661 (2013).
36. Seo, D.H. *et al.* First-principles study on lithium metal borate cathodes for lithium rechargeable batteries. *Phys. Rev. B* **83**, 205127 (2011).
37. Bondareva, O. S., Simonov, M. A., Egorov-Tismenko, Y. K. & Belov, N. V. Crystal structure of $LiZn[BO_3]$ and $LiMn[BO_3]$. *Kristallografiya* **23**, 487-490 (1978).
38. Legagneur, V. *et al.* $LiMBO_3$ (M=Mn, Fe, Co): synthesis, crystal structure and lithium deinsertion/insertion properties. *Solid State Ionics* **139**, 37-46 (2001).
39. Piffard, Y., Rangan, K. K., An, Y., Guyomard, D. & Tournoux, M. Cobalt Lithium Orthoborate, $LiCoBO_3$. *Acta Crystallographica C* **54**, 1561-1563 (1998).
40. Islam, M. M., Bredow, T. & Heitjans, P. Formation and Mobility of Li Point Defects in $LiBO_2$: A First-Principles Investigation. *J. Phys. Chem. C* **115**, 12343-12349 (2011).
41. Kirfel, A., Will, G. & Stewart, R. F. The chemical bonding in lithium metaborate, $LiBO_2$. Charge densities and electrostatic properties. *Acta Crystallographica B* **39**, 175-185 (1983).
42. Meng, F. H. *et al.* (in prepare)
43. Shi, S. Q., Qi, Y., Li, H. & Hector, L. G. Defect Thermodynamics and Diffusion Mechanisms in Li_2CO_3 and Implications for the Solid Electrolyte Interphase in Li-Ion Batteries. *J. Phys. Chem. C* **117**, 8579-8593 (2013).
44. Idemoto, Y., Ricardson, J. W. Jr., Koura, N., Kohara, S. & Loong, C.-K. Crystal structure of $(Li_xK_{1-x})_2CO_3$ (x = 0, 0.43, 0.5, 0.62, 1) by neutron powder diffraction analysis. *J. Phys. Chem. Solids* **59**, 363-376 (1998).
45. Zhang, Y., Zhao, Y. S. & Chen, C. F. Ab initio study of the stabilities of and mechanism of superionic transport in lithium-rich antiperovskites. *Phys. Rev. B* **87**, 134303 (2013).
46. Zhao, Y. S. & Daemen, L. L. Superionic Conductivity in Lithium-Rich Anti-Perovskites. *J. Am. Chem. Soc.* **134**, 15042-15047 (2012).
47. Du, Y. A. & Holzwarth, N. A. W. Mechanisms of Li^+ diffusion in crystalline γ- and β-Li_3PO_4 electrolytes from first principles. *Phys. Rev. B* **76**, 174302 (2007).
48. Keffer, C., Mighell, A. D., Mauer, F., Swanson ,H. & Block, S. Crystal structure of twinned low-temperature lithium phosphate. *Inorganic Chemistry* **6**, 119-125 (1967).
49. Yakubovich, O. V. & Urosova, V. S. Electron distribution in lithiophosphatite: Crystallochemical features of orthophasphates with hexagonal close packing. *Kristallografiya* **42**, 301-308 (1997).
50. Lepley, N. D., Holzwarth, N. A. W. & Du, Y. A. Structures, Li^+ mobilities, and interfacial properties of solid electrolytes Li_3PS_4 and Li_3PO_4 from first principles. *Phys. Rev. B*, **88**, 104103 (2013).
51. Homma, K. *et al.* Crystal structure and phase transitions of the lithium ionic conductor Li_3PS_4. *Solid State Ionics* **182**, 53-58 (2011).
52. Xiong, K., Longo, R. C., Santosh, K. C., Wang, W. & Cho, K. Behavior of Li defects in solid

electrolyte lithium thiophosphate $Li_7P_3S_{11}$: A first principles study. *Comput. Mater. Sci.* **90**, 44-49 (2014).

53. Yamane, H. *et al.* Crystal structure of a superionic conductor, $Li_7P_3S_{11}$. *Solid State Ionics* **178**, 1163-1167 (2007).
54. Clark, J. M. *et al.* High voltage sulphate cathodes $Li_2M(SO_4)_2$ (M = Fe, Mn, Co): atomic-scale studies of lithium diffusion, surfaces and voltage trends. *J. Mater. Chem. A* **2**, 7446-7453 (2014).
55. Reynaud, M. *et al.* $Li_2Fe(SO_4)_2$ as a 3.83 V positive electrode material. *Electrochem. Commun.* **21**, 77-80 (2012).
56. Reynaud, M., Rousse, G., Chotard, J. N., Rodriguez-Carvajal, J. & Tarascon, J. M. Marinite $Li_2M(SO_4)_2$ (M = Co, Fe, Mn) and $Li_1Fe(SO_4)_2$: Model Compounds for Super-Super-Exchange Magnetic Interactions. *Inorg. Chem.* **52**, 10456-10466 (2013).
57. Jalem, R., Nakayama, M. & Kasuga, T. Lithium ion conduction in tavorite-type $LiMXO_4F$ (M–X: AlP, MgS) candidate solid electrolyte materials. *Solid State Ionics* **262**, 589-592 (2014).
58. Simonov, V. I. & Belov, N. V. The determination of the structure of amblygonite by the Minimum-Function Method. *Kristallografiya* **3**, 429-438 (1958).
59. Sebastian, L., Gopalakrishnan, J. & Piffard, Y. Synthesis, crystal structure and lithium ion conductivity of $LiMgFSO_4$. *J. Mater. Chem.* **12**, 374-377 (2002).
60. Tripathi, R., Gardiner, G. R., Islam, M. S. & Nazar, L. F. Alkali-ion Conduction Paths in $LiFeSO_4F$ and $NaFeSO_4F$ Tavorite-Type Cathode Materials. *Chem. Mater.* **23**, 2278-2284 (2011).
61. Melot, B. C. *et al.* Magnetic Structure and Properties of the Li-Ion Battery Materials $FeSO_4F$ and $LiFeSO_4F$. *Chem. Mater.* **23**, 2922-2930 (2011).
62. Mueller, T., Hautier, G., Jain, A. & Ceder, G. Evaluation of Tavorite-Structured Cathode Materials for Lithium-Ion Batteries Using High-Throughput Computing. *Chem. Mater.* **23**, 3854-3862 (2011).
63. Lu, X. *et al.* (in prepare)
64. Aatiq, A., Menetrier, M., Croguennec, L., Suard, E. & Delmas, C. On the structure of $Li_3Ti_2(PO4)_3$. *J. Mater. Chem.* **12**, 2971-2978 (2002).
65. Kang, K., Morgan, D. & Ceder, G. First principles study of Li diffusion in I-Li_2NiO_2 structure. *Phys. Rev. B* **79**, 014305 (2009).
66. Rieck, H. & Hoppe, R. Ein neues Oxoniccolat: Li_2NiO_2. *Zeitschrift fuer Anorganische und Allgemeine Chemie* **392**, 193-196 (1972).
67. Islam, M. M., Bredow, T. & Minot, C. Theoretical Analysis of Structural, Energetic, Electronic, and Defect Properties of Li_2O. *J. Phys. Chem. B* **110**, 9413-9420 (2006).
68. Farley, T.W.D., Hayes, W., Hull, S., Hutchings., M. T. & Vrtis, M. Investigation of thermally induced Li^+ ion disorder in Li_2O using neutron diffraction. *J. Phys.: Condens. Matter* **3**, 4761-4781 (1991).
69. Chen, Y. C., Ouyang, C. Y., Song, L. J. & Sun, Z. L. Electrical and Lithium Ion Dynamics in Three Main Components of Solid Electrolyte Interphase from Density Functional Theory Study. *J. Phys. Chem. C* **115**, 7044-7049 (2011).
70. Streltsov, V. A., Tsirelson, V. G., Ozerov, R. P. & Golovanov, O. A. Electron and Thermal Parameters of Ions in CaF_2. Results of the Application of the Regularized Least Squares Method. *Kristallografiya* **33**, 90-97 (1988).
